# The Influence of Polysaccharides on the Textural Properties and Water Retention Capacity of Animal–Plant Dual-Protein Gels

**DOI:** 10.3390/gels12010040

**Published:** 2025-12-31

**Authors:** Wenhao Gao, Zhiming Wang, Zhihao Zhao, Yuanyuan Deng, Jingjing Wang, Pengfei Zhou, Ping Li, Yan Zhang, Mingwei Zhang, Guang Liu

**Affiliations:** 1 College of Life Sciences, Yangtze University, Jinzhou 430070, China; gwh143019@163.com; 2 Sericultural & Agri-Food Research Institute Guangdong Academy of Agricultural Sciences, Key Laboratory of Functional Foods, Ministry of Agriculture and Rural Affairs, Guangdong Key Laboratory of Agricultural Products Processing, Guangzhou 510610, China; fezmwang@163.com (Z.W.); zhaozhihao1991@163.com (Z.Z.); yuanyuan_deng@sohu.com (Y.D.); zhoupengfei@gdaas.cn (P.Z.); liping2019@gdaas.cn (P.L.); zhangyanhb@tom.com (Y.Z.); 3 Department of Food Science, Foshan University, Foshan 528000, China; jjwang@fosu.edu.cn; 4 Food Laboratory of Zhongyuan, Luohe 462300, China

**Keywords:** pork–whole soy milk gel, polysaccharides, dysphagia, texture modification, IDDSI

## Abstract

To develop nutrient-rich whole-food gels for individuals with dysphagia, this study constructed a pork–whole soy milk composite gel (PSG) using a hybrid animal–plant protein approach. The effects of xanthan gum, konjac glucomannan, and guar gum at different concentrations (0.5%, 1.0%, and 1.5%) on the gel properties, protein conformation, and microstructure of different PSGs were systematically investigated. The results indicated that polysaccharides interfered with protein cross-linking and disrupted the gel network, leading to reduced gel hardness. Due to their abundant hydrophilic groups, the polysaccharides significantly enhanced the water-holding capacity (*p* < 0.05), achieving a synergistic outcome of structural softening and functional reinforcement. A comprehensive evaluation identified the PSG with 1.0% xanthan gum as the optimal formulation, which exhibited a 43.2% increase in water-holding capacity and a hardness only 23.5% of the control, complying with both International Dysphagia Diet Standardisation Initiative (IDDSI) Level 5 and Japanese Dysphagia Diet Level III standards. This study elucidates the mechanism by which polysaccharides modulate whole-food protein gels and provides a practical strategy for developing dysphagia-friendly foods that preserve nutritional quality and are suitable for industrial production.

## 1. Introduction

The global population aged 65 and above is projected to rise from 9.3% in 2020 to around 16% by 2050, highlighting the urgent need to ensure adequate nutrition and health in older adults [1]. Dysphagia, prevalent among the elderly, elevates risks of malnutrition, dehydration, depression, pneumonia, and choking [2]. Developing nutrient-rich foods with modified texture is therefore essential to improve the quality of life for individuals with swallowing disorders [3].

The diet for swallowing disorders is typically categorized into three types: thickened liquids, pureed foods, and gel-based foods. Although thickened liquids and pureed foods are the most used, they often present challenges such as insufficient nutritional density, poor sensory appeal, and lack of convenience. In contrast, gels are moist, soft, visually appealing, and easy to manipulate in the mouth [4]. These attributes help enhance patients’ appetite, making gel-based foods an increasingly important focus in the research and development of foods for individuals with swallowing disorders. Current research primarily focuses on developing texture-adjustable gel systems based on single animal or plant proteins supplemented with various polysaccharides. By modulating protein–polysaccharide types and ratios, these systems can produce gels with adjustable textures that precisely meet different International Dysphagia Diet Standardisation Initiative (IDDSI) [5] grading requirements. For example, adjusting the proportion of rice starch and soy protein in a composite gel can yield texture-modified foods tailored to patients with varying degrees of swallowing difficulties [6]. Similarly, a ternary composite gel system composed of bovine tendon collagen, cassava starch, and Polygonatum sibiricum polysaccharide (PSP) exhibits high water-holding capacity. By adjusting the amount of PSP, this gel can satisfy IDDSI standards at various levels [7].

However, relying on a single protein source may increase digestive and absorption demands, as well as lead to nutritional imbalances in elderly. Combining animal and plant proteins can improve amino acid profiles and achieve better nutritional balance [8]. Recent studies have looked into developing dysphagia foods using mixed animal and plant protein formulations. Lin et al. [9] showed that adding plant proteins enhances the nutritional value of surimi products and improves the microstructure of surimi gels. This results in denser, more uniform gels with greater strength. Lee et al. [3] found that the pea protein-surimi mix composite gel containing more than 60% pea protein has a suitable texture for individuals with swallowing difficulties and increases the in vitro digestibility of the gel. Despite these advances, composite gel foods based on extracted and isolated proteins suffer from drawbacks such as poor taste, high development costs, and lack of nutritional components. Therefore, the direct use of whole food as raw materials for the preparation of gel food, more in line with the nutritional needs of patients with swallowing difficulties.

Whole foods (meat and legumes) have richer flavors and contain more other nutrients compared to colloids made only from isolated proteins and polysaccharides [10,11]. Pork and soybean products are among the largest sources of animal and plant protein; their relatively low cost and easy processing make them more conducive to large-scale industrial production. Pork is rich in high-quality protein, B vitamins, and essential minerals such as zinc and iron [12]. Soybean products, in addition to providing high-quality vegetable protein, also have bioactive isoflavones and saponins [13]. Therefore, mixing pork and whole soy milk to develop a double-protein whole food matrix for dysphagia gel food can provide more options for patients with dysphagia. Furthermore, studies have shown that blending soy protein with pork protein can improve the gel properties of meat products and enhance their water-holding capacity, supporting the feasibility of pork–whole soy milk composite gels (PSGs) [14].

In the preliminary work, it was found that the composite gel system containing only whole soy milk and pork exhibited issues such as excessive hardness, inadequate water retention, and high cooking loss. The investigation revealed that polysaccharides could enhance the performance of composite gels. The incorporation of xanthan gum (XG) enhances the rheological properties, self-supporting capacity, and 3D printing ability of pork pastes while improving the water absorption rate [15]. The inulin–konjac glucomannan (KGM) mixture interacts with fish myofibrillar proteins, improving swallowing performance, enhancing water retention, and effectively counteracting the inherent fishy odor of the raw material, thereby elevating the overall sensory experience [3]. The addition of guar gum (GG) significantly improves the water retention capacity and textural properties of soybean curd [16].

Therefore, the present study prepared composite gel system from soybeans and pork, and the effects of three polysaccharides (XG, KGM, GG) on water-holding capacity, water distribution, color difference, texture properties, rheological properties, and IDDSI swallowing grade of composite gels were systematically evaluated. The underlying mechanisms were elucidated through microstructural analysis, secondary structure changes, and molecular interactions. The findings provide technical guidance for developing dysphagia-friendly foods using whole-component systems.

## 2. Results and Discussion

### 2.1. Effects of Different Polysaccharides on the Water Properties of PSGs

#### 2.1.1. Cooking Loss and Water-Holding Capacity

**Figure 1:**
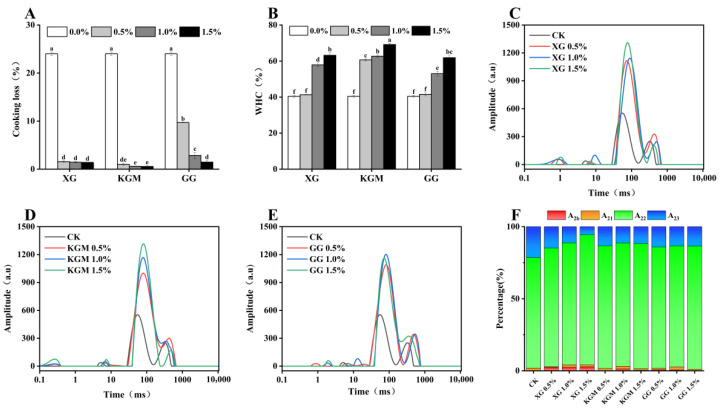
Cooking loss (**A**), water holding capacity (**B**), T2 spectrum (**C**–**E**), and water distribution (**F**) of PSG with distinct formulations. Different lowercase letters in the same category indicate significant differences (*p* < 0.05).

Heat-induced gelation of proteins often leads to structural deformation and subsequent water migration and exudation, which is quantified as cooking loss and serves as a critical indicator of product stability [17]. Adding polysaccharides can effectively reduce this loss. As shown in Figure 1A, the cooking loss of PSG incorporated with XG, KGM, or GG decreased significantly (*p* < 0.05) with increasing polysaccharide concentration. Both XG and KGM lowered cooking loss to below 1.5% at additions ≥ 0.5%, compared to 24% in the control. Notably, 1.5% KGM performed best, with only 0.6% loss, indicating nearly complete water retention. In contrast, at a low concentration (0.5%), GG resulted in a cooking loss of 9.7%, which was significantly higher than that of XG and KGM at the same level. However, increasing the GG concentration to 1.5% also reduced the cooking loss to 1.5%. Overall, the efficacy of the polysaccharides in mitigating cooking loss followed the order: KGM > XG > GG.

WHC is a necessary index to evaluate the food quality of dysphagia patients, which is related to the moisture and juiciness of food. Superior WHC enhances the texture and lubrication of food, thereby reducing the resistance of food through the esophagus and stomach [6]. Consistent with the cooking loss results, the WHC of the composite gels was significantly improved (*p* < 0.05) by the incorporation of all three polysaccharides in a concentration-dependent manner Figure 1B. The enhancement effect also followed the same order: KGM > XG > GG. These findings align with previous reports that polysaccharide composite gels generally exhibit superior water retention properties [18]. The improved WHC is primarily attributed to the abundant hydrophilic groups (e.g., hydroxyl and carboxyl groups) in polysaccharide molecules, which strongly bind water via hydrogen bonding, thereby immobilizing more water within the gel network [19,20]. The superior performance of KGM over XG and GG can be reasonably attributed to its inherently stronger water-absorbing capacity and superior thermal stability, which allow it to form and maintain a more robust hydrated network during the heating process [21]. Furthermore, it has been suggested that the hydrophilic and hydration capabilities of polysaccharides can enhance WHC independently of network compactness [22], and synergistic interactions between proteins and polysaccharides may also contribute to improved water retention [23].

In conclusion, XG, KGM, and GG significantly enhanced the cooking loss and water retention of PSG through their pronounced hydrophilic properties. KGM and XG were particularly effective at higher concentrations.

#### 2.1.2. Water Distribution and Binding States in PSGs Analyzed by LF-NMR

To understand how polysaccharides affect water distribution in composite gels, the transverse relaxation time (T_2_) of various gel samples was analyzed using low-field nuclear magnetic resonance (LF-NMR). As depicted in Figure 1C–E, the T_2_ relaxation spectra of the composite gels exhibited four distinct peaks, representing different water states: T_2b_(0.1 ms–1 ms), T_21_(1 ms–10 ms), T_22_(10 ms–100 ms), T_23_(100 ms–1000 ms). Among these, T_2b_ and T_21_ represent water tightly bound to macromolecules such as proteins in the gel matrix [6], while T_22_ corresponds to immobilized water trapped within the gel network. Figure 1F shows the variation in the peak area ratio (A_2_) of the sample amplitude. The relative peak area of T_22_ exceeded 76% in all samples, indicating that immobilized water constitutes the dominant form of moisture in the composite gels. The T_23_ peak is attributed to more mobile free water.

Compared with the control group, the addition of polysaccharides significantly increased (*p* < 0.05) the proportion of immobilized water and decreased the content of free water. Notably, XG induced a more pronounced redistribution of water states than KGM or GG, leading to a greater reduction in free water and a stronger enhancement of immobilized water at equivalent concentrations. The results show that all three polysaccharides effectively promote the formation of bound water and inhibit the presence of free water, which is attributed to the strong hydration capacity of the mid-polar groups in polysaccharides. This aligns with the observed improvement in water retention. In summary, polysaccharides optimize water distribution in PSG by increasing bound water and reducing free water, thereby enhancing water retention. This enhancement in water retention positively affects foods intended for individuals with swallowing disorders.

### 2.2. Effects of Different Polysaccharides on the Color of PSGs

Color is a critical visual attribute that directly influences consumer acceptance of food products [24]. As shown in Table 1, the incorporation of polysaccharides significantly alters the color parameters of the composite gel. Compared to the control group, a significant increase in the L* (lightness) and W (whiteness) values of the gel was observed only with the addition of 1.5% XG (*p* < 0.05). No statistically significant differences in these color parameters were found for gels containing either KGM or GG at any of the tested concentrations, nor for XG at concentrations of 0.5% and 1.0%, when compared to the control (*p* > 0.05). Polysaccharide addition also influenced the a* values, showing an overall decreasing trend, which indicates a reduction in the redness of the PSG. With increasing XG concentration, the b* value exhibited a continuous decline, whereas KGM and GG demonstrated an initial increase followed by a decrease in b* values. To comprehensively assess the overall visual impact of these color modifications, the total color difference (ΔE) between each polysaccharide added sample and the control was calculated. The gel containing 1.5% XG showed a ΔE value of 2.79, which exceeds the threshold of 2.0, indicating a potentially perceptible difference to an average observer. For samples with 1.0% XG, 0.5% KGM, and 0.5% GG, the ΔE values fell within the range of 1.0 < ΔE < 2.0, suggesting that the color differences would be discernible only to trained observers under close comparison. All remaining samples exhibited ΔE values below 1.0, representing differences that are generally imperceptible to the human eye [25].

In summary, polysaccharide addition effectively modifies the color of the PSG system. This finding aligns with previous studies, which suggest that polysaccharides can regulate gel color through cross-linking interactions with protein molecules [26], Among the three polysaccharides tested, XG most significantly enhanced the lightness and whiteness of the gels. This performance aligns more closely with consumers’ visual preferences, suggesting greater potential for market acceptance.

### 2.3. Effects of Different Polysaccharides on the Chewing and Swallowing Function of PSGs

#### 2.3.1. TPA

The texture of gelled foods is a key determinant of sensory quality and swallowing performance [27]. In this study, hardness, adhesiveness, and agglomeration of the samples were evaluated. The swallowing suitability of the composite gels was classified according to the Japanese Care Food Association standards, which specify the following thresholds for dysphagia-friendly foods: hardness < 2 × 10^4^ N/m^2^, adhesiveness < 1.5 × 10^3^ N/m^2^, and agglomeration between 0.2 and 0.9.

As summarized in Table 2, the addition of XG, KGM, and GG results in a significant reduction in gel hardness (*p* < 0.05), demonstrating concentration dependence. The hardness values of PSGs with all added polysaccharides are below the standard threshold of 2 × 10^4^ N/m^2^ for foods intended for individuals with swallowing disorders in Japan. Among these, the gel hardness of 1% and 1.5% XG is the lowest, measuring only 0.23 times that of the PSG in the control group. The pronounced softening effect of XG on protein gels aligns with the findings of Pemattileke et al. [28], who reported that among 14 tested hydrophilic colloids, XG reduced the hardness of beef protein gel to 2% of the control group, yielding the softest texture. Polysaccharide addition also significantly influenced adhesiveness. XG notably reduced adhesiveness (*p* < 0.05), indicating that less force is required to form a cohesive bolus during mastication. In contrast, KGM initially increased and then decreased adhesiveness, yet all KGM-containing gels retained relatively high values, suggesting greater chewing effort. Although GG at 0.5% significantly reduced adhesiveness, it still exceeded the Japanese standard limit (<1.5 × 10^3^ N/m^2^). Only the XG 1.0% and XG 1.5% formulations fully met the adhesiveness requirement. Furthermore, agglomeration increased with polysaccharide concentration and exceeded that of the control in all cases. All samples fell within the acceptable Japanese standard range (0.2–0.9), indicating that polysaccharides enhance internal binding within the gel matrix. This improved agglomeration helps prevent excessive breakdown during chewing, promoting safer swallowing. The softening of the composite gel is attributed to the alteration of the protein gel network by polysaccharides through the mechanisms of spacing effect and competing adsorption of water and polysaccharides [29]. Such texture modification is beneficial, as high-viscosity or hard foods are not suitable for older adults or individuals with dysphagia [30].

In conclusion, based on the texture profile analysis, only the composite gels with 1.0% and 1.5% XG exhibit suitably low hardness and adhesiveness, coupled with appropriate agglomeration, fully satisfying the Grade III dysphagia food criteria outlined by the Japanese standard.

#### 2.3.2. IDDSI

The IDDSI framework provides a systematic and scientifically validated approach for classifying food textures based on swallowing safety, using a scale from 0 to 7 [5]. In this study, the fork pressure test and spoon tilt test were employed to further evaluate the suitability of PSGs as dysphagia-friendly foods. As summarized in Table 3, most composite gels particularly those containing XG 0.5%, KGM (0.5–1.5%), and GG (0.5–1.5%) were classified as Level 6 (Soft & Bite-Sized). In contrast, gels with 1.0% and 1.5% XG were categorized as Level 5 (Minced & Moist). The control group did not fall within any IDDSI category. In the fork pressure test, all polysaccharide-containing samples were easily compressed and did not recover their original shape after fork removal—a characteristic of softer texture categories. When subjected to thumb pressure, Level 6 samples exhibited visible nail blanching, whereas no such blanching occurred with XG 1.0% and 1.5% gels, confirming their softer consistency. In the spoon tilt test, all composite gels slid smoothly without residue, indicating appropriate agglomeration and low adhesion.

These IDDSI findings are consistent with the instrumental texture data. Collectively, they demonstrate that polysaccharide addition effectively modulates the texture of PSGs, reducing hardness and adhesiveness to levels suitable for dysphagia diets. Notably, XG at 1.0% and 1.5% concentrations shows the greatest potential for developing compliant dysphagia foods, meeting both the Level 5 products.

### 2.4. Effects of Different Polysaccharides on the Rheological Properties of PSGs

#### 2.4.1. Frequency Sweep Test

**Figure 2:**
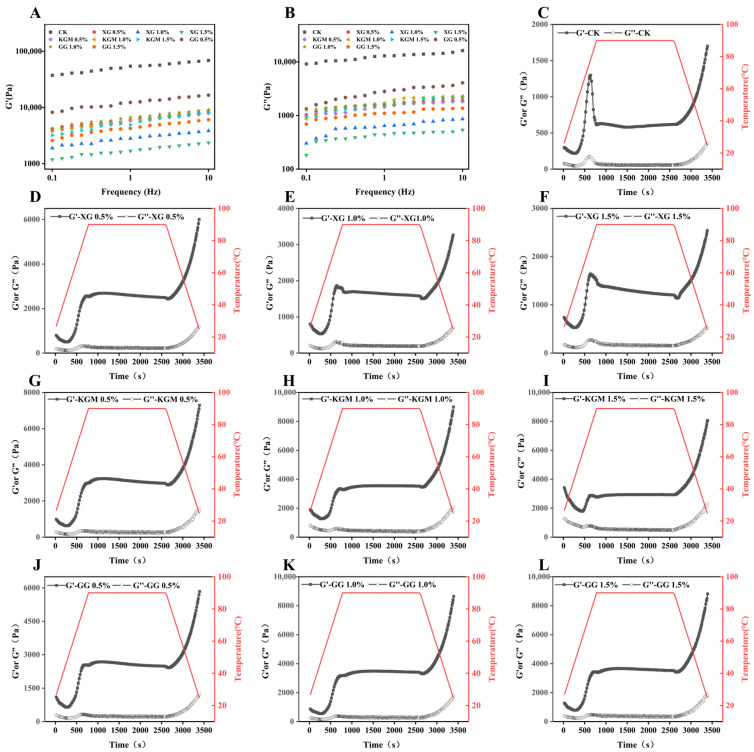
Rheological properties of PSGs with different formulations. (**A**): Storage modulus (G’), (**B**): loss modulus (G”). The temperature sweep of PSGs with different formulations (**C**–**L**).

The rheological behavior of food gels is critical for evaluating their textural and swallowing characteristics, particularly in the development of dysphagia-friendly products [31]. The storage modulus (G′) reflects the elastic behavior and structural strength of the gel, while the loss modulus (G″) represents its viscous flow properties. As shown in Figure 2A,B, G′ consistently exceeded G″ throughout the frequency range, with tan δ (G″/G′) remaining below 1. This behavior confirms that all gels exhibited solid-dominant viscoelasticity, aligning with the rheological requirements for dysphagia-oriented foods and suggesting their suitability for safe consumption by older adults [7]. Polysaccharide addition significantly reduced G′ (*p* < 0.05), indicating gel softening in a concentration-dependent manner. XG at 1% and 1.5% showed the most pronounced reduction, consistent with texture profile results. This softening may be attributed to electrostatic repulsion and steric hindrance introduced by polysaccharides, which inhibit excessive protein aggregation and promote a more open network structure.

To further analyze the gel network, the power-law model (log(G’) = n × log(ω) + k) was applied. A higher slope (n) suggests a structure dominated by non-covalent cross-links, while the intercept (k) reflects the strength of molecular interactions within the gel. As summarized in Table 4, all polysaccharide added PSGs showed increased n values and decreased k values compared to the control, indicating enhanced non-covalent cross-linking and weakened molecular interaction forces. For XG-PSGs, the n value increased initially and then decreased with concentration yet remained above the control. In contrast, KGM-PSGs and GG-PSGs exhibited a continuous decrease in n. The decline in k was most evident in XG-PSGs. Since stronger molecular interactions (higher k) correlate with better viscoelasticity [32,33], the observed decrease in k aligns with the softened gel behavior.

#### 2.4.2. Temperature Sweep Test

The gelation process was investigated by monitoring G′ and G″ during heating, holding at 90 °C, and cooling Figure 2C–L. The gelation temperature, defined as the temperature at which G′ begins to increase sharply [34], was found to rise with increasing polysaccharide concentration Table 3. This suggests that polysaccharides may inhibit protein cross-linking, thereby delaying network formation. Notably, at equivalent concentrations, XG-PSGs exhibited the lowest gelation temperature, indicating that XG facilitates gel formation with lower energy input compared to KGM or GG. Throughout the temperature sweep, G′ remained higher than G″ without crossover, consistent with the behavior of previously reported swallow-safe inulin/KGM-protein gels [4]. During initial heating, G′ was higher in polysaccharide- containing PSGs than in the control, likely due to increased mixture viscosity enhancing system stability. As temperature rose, both moduli increased, with G′ rising more sharply, indicating the formation of a three-dimensional protein network. During the 90 °C holding stage, both G′ and G″ continued to increase, and a significant reinforcement of the gel network occurred upon cooling. To gain further insight into the cross-linking behavior during cooling, the ratio G′_25°C_/G′_90°C_ was calculated [33]. A higher ratio indicates the dominance of non-covalent bonds (e.g., hydrogen bonding, electrostatic interactions), whereas a lower ratio suggests a greater contribution from covalent bonds (e.g., disulfide bonds) or hydrophobic interactions. As shown in Table 4, all PSGs exhibited relatively low G′_25°C_/G′_90°C_ ratios, indicating that gel formation is primarily driven by covalent and/or hydrophobic interactions. The ratios further decreased in polysaccharide-added groups, implying that polysaccharides promote the involvement of disulfide bonds or hydrophobic interactions during network formation.

In summary, rheological analysis shows that XG, KGM, and GG significantly affect the viscoelastic properties and gelation of PSGs by reducing molecular interaction forces, modifying cross-linking mechanisms, and raising gelation temperatures.

### 2.5. Mechanism of Polysaccharides in Regulating PSGs Structure

#### 2.5.1. Molecular Forces

**Figure 3:**
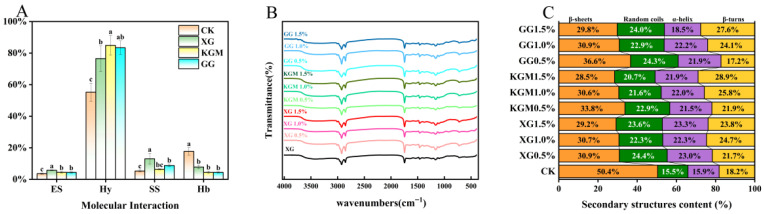
Molecular Interaction (**A**) FTIR spectra (**B**) and protein secondary structure (**C**) of different formulations of PSGs.

To further elucidate how polysaccharides regulate the gel properties of PSGs, we analyzed the molecular interaction forces in selected samples (CK, XG 1.0%, KGM 1.0%, and GG 1.0%) using a chemical extraction method with four different protein interaction-targeting reagents. As shown in Figure 3A, electrostatic interactions were relatively weak in all samples, indicating they were not the primary driving force for protein network formation in this study. Instead, hydrophobic interactions played the dominant role in stabilizing the gel structure.

During polysaccharide incorporation, hydrophobic interactions were further enhanced while hydrogen bonding significantly weakened. This shift aligns with previous reports that stronger hydrophobic interactions (typically accompanied by reduced hydrogen bonding) result in softer protein–polysaccharide composite gels [35], consistent with the decreased gel hardness observed in our study. However, excessively strong hydrophobic interactions may promote protein aggregation, leading to a coarser network structure [4]. Such aggregation could concurrently facilitate an increase in disulfide bond formation. The reduction in intermolecular hydrogen bonds after polysaccharide addition is likely attributed to competitive binding between polysaccharides and proteins, which diminishes the formation of protein–protein and protein–water hydrogen bonds. Despite this, the water-holding capacity improved, suggesting that polysaccharides rather than proteins became the primary factor for water retention in this system. Furthermore, the increases in disulfide bonds and hydrophobic interactions correspond with the lower G′_25_ °C/G′_90_ °C ratio obtained from rheological analysis, indicating that covalent bonds and hydrophobic forces contributed more significantly to the formation of the final gel network.

In summary, polysaccharide incorporation effectively modulated the types and intensities of molecular forces within PSGs, specifically enhancing hydrophobic interactions and disulfide bonds while weakening hydrogen bonding. These changes not only influenced the gelation process but ultimately determined the structural characteristics of PSGs.

#### 2.5.2. Secondary Structure of Proteins

FTIR spectroscopy was used to investigate changes in protein secondary structure, to elucidate how polysaccharides regulate composite gel texture. Figure 3B shows the FTIR spectra of PSGs prepared with different types and concentrations of polysaccharides. The overall FTIR spectra of PSG samples prepared with varying polysaccharide types and concentrations showed no significant differences, suggesting the absence of covalent interactions between the polysaccharides and proteins. Deconvolution of the amide I band (1600–1700 cm^−1^) was performed to quantify the proportions of protein secondary structures. The calculated protein secondary structure contents are presented in Figure 3C. The results indicated that the content of β-sheets gradually decreased from 50.4% to approximately 30% with increasing polysaccharide concentration, exhibiting a clear concentration-dependent trend. Concurrently, the proportions of α-helices, β-turns, and random coils slightly increased. This structural rearrangement can be attributed to polysaccharides competing with proteins for hydrogen bonds, combined with steric hindrance and changes in the electrostatic environment. These effects collectively disrupt the hydrogen-bonded network between β-sheet layers, leading to a reduction in β-sheet content and an increase in structural disorder. These findings are consistent with previous reports that polysaccharide addition interferes with the formation of intermolecular β-sheets, resulting in a looser gel network and reduced gel strength [36].

In summary, FTIR analysis reveals that polysaccharides disrupt the β-sheet-dominated hydrogen-bonding network in proteins via competitive hydrogen bonding and steric effects, promoting a shift toward more disordered conformations. This molecular-level change explains the observed reduction in gel strength.

#### 2.5.3. Microstructure

**Figure 4:**
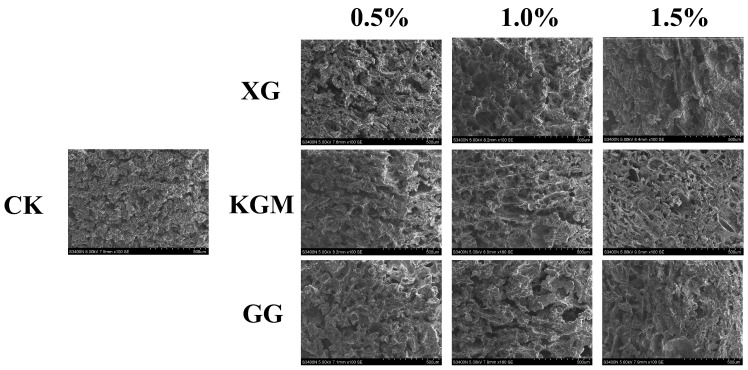
SEM micrographs of PSG with different formulations.

SEM was used to further examine the microstructure of the composite gels. As shown in Figure 4, the control PSG exhibited a dense and uniform network of protein fibers, characterized by evenly distributed micropores, small pore size, and high structural continuity. In contrast, the incorporation of XG, KGM, or GG resulted in a noticeably looser network structure. The protein fiber bundles appeared “diluted” or fragmented, accompanied by a significant increase in pore number, enlarged pore size, thinner pore walls, and localized structural collapse. These structural changes were most pronounced in the XG-containing gels. The resulting “large pore–thin wall” morphology contributed to the reduction in mechanical strength, as indicated by the significant decrease in hardness—a finding consistent with the texture profile analysis.

This microstructural trend aligns with previous reports, such as the pore-enlargement effect of excessive CMC [37] and the network non-compaction observed in κ-carrageenan/gelatin systems beyond a critical polysaccharide content [38]. The underlying mechanism can be attributed to the rigid backbones of polysaccharides, which introduce steric hindrance between protein cross-linking sites, thereby weakening protein–protein interactions. Additionally, in the case of XG, electrostatic repulsion between its anionic side chains and negatively charged protein surfaces further disrupts network integrity. This may explain why XG induced a more pronounced softening effect compared to the neutral polysaccharides KGM and GG.

Notably, despite the structural loosening, all three polysaccharides enhanced the functional properties of the gels through their abundant hydrophilic groups. This illustrates a “structure-weakening yet function-enhancing” mechanism, providing a clear microstructural basis for the rational design of dysphagia gels with improved water-retention capacity.

### 2.6. Discussion

**Figure 5:**
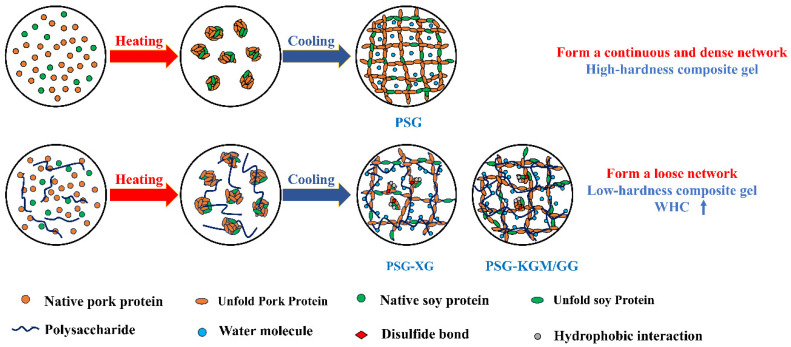
Mechanism of Formation of Pork-Whole Soy Milk Protein and Polysaccharide Composite Gel.

The present study demonstrates that the incorporation of XG, KGM, and GG significantly modulates the texture, water retention, and structural properties of PSGs. As illustrated in Figure 5, a schematic model is presented to elucidate the formation mechanism of the gel network in the pork–soymilk system, both with and without polysaccharides. Specifically, all three polysaccharides reduced gel hardness, which can be attributed to a decrease in the relative content of β-sheets in the protein secondary structure and a shift in intermolecular forces—notably, an enhancement in hydrophobic interactions and a reduction in hydrogen bonding. These molecular-level changes were corroborated by microstructural observations, which revealed a looser gel network in polysaccharide-modified PSGs, further supporting the decrease in mechanical strength. The resulting composite gels passed the IDDSI fork pressure test, confirming their suitability as dysphagia-friendly foods for older adults. Moreover, the abundant hydrophilic groups in the polysaccharides contributed to a lower cooking loss and an increase in immobilized water, thereby enhancing the WHC of the gels.

Conventional studies often report that polysaccharides improve water retention by forming a denser network that traps water. However, such structural densification is typically accompanied by an increase in hardness, creating a trade-off between texture and water retention. In contrast, the polysaccharides used in this study simultaneously enhanced WHC and reduced hardness—a functional combination that aligns more effectively with the sensory and safety requirements of foods designed for populations with swallowing difficulties.

Although XG, KGM, and GG influenced PSG properties in a generally consistent direction, XG induced a more pronounced reduction in hardness at equivalent concentrations. This can be explained by the anionic nature of XG, which contains carboxyl groups (–COOH), in contrast to the neutral hydroxyl-dominated structures of KGM and GG. The charged groups in XG likely introduce stronger electrostatic interference with protein–protein cross-linking, enhance 3hydration, and promote phase separation, collectively leading to a more open gel network and a greater softening effect.

## 3. Conclusions

This study demonstrates that the incorporation of xanthan gum (XG), konjac glucomannan (KGM), and guar gum (GG) effectively modulates the properties of pork–whole soy milk composite gel (PSG), making it suitable as a food for individuals with swallowing difficulties. The addition of polysaccharides enhances the gel’s water retention via their hydrophilic groups and alters protein interactions within the PSG, leading to a reduction in β-sheet structures in the protein secondary conformation. Consequently, gel hardness decreases upon polysaccharide addition, with the softening effect being most pronounced for the anionic polysaccharide XG. The 1.0% XG is the optimal choice to achieve the suitable hardness and adhesion required for safe swallowing (Japanese grade III/IDDSI grade 5) while providing excellent water retention, agglomeration performance, and balanced industrial cost-effectiveness, which can be used to improve the safety of food processing. This work not only elucidates the mechanism by which polysaccharides tailor the pork–whole soy milk composite gel network but also identifies 1.0% XG as the recommended dosage for developing PSG-based dysphagia foods with superior texture, safety, and consumer acceptability.

## 4. Materials and Methods

### 4.1. Materials

Pork loin (containing 23.19% protein and 6.59% fat) and pork backfat (containing 1.51% protein and 89.23% fat) were procured from a local supermarket. Hulled soybeans (containing 37.98% protein and 20.87% fat) were sourced from Jilang Rice Industry Co., Ltd. (Qiqihar, China). Xanthan gum was provided by Xinjiang Meihua Amino Acid Co., Ltd. (Wujiaqu, China). Konjac glucomannan and Guar gum were purchased from Jiahe Xuri Co., Ltd. (Shenzhen, China).

### 4.2. Preparation of Pork Mince and Whole Soy Milk

Minced pork: Loin (with visible connective tissue and fat removed) was combined with back fat at a mass ratio of 8:2 and minced once through a 6 mm plate (MJ-LZ225, Midea, Foshan, China).

Whole soy milk: Dehulled soybeans were soaked in deionized water (1:10, *w*/*v*) for 12 h at 4 °C, drained, and processed in a soymilk maker (DJ12X-365, Joyoung, Shanghai, China) using the integrated grind-and-boil cycle. The resulting slurry was passed through a JM-L colloid mill (Wenzhou Qiangzhong Machinery Technology Co., Ltd., Wenzhou, China) for 30 min at ambient temperature to obtain a homogeneous dispersion. The particle size was confirmed to be <100 µm using a Betteisize 2600 laser particle size analyzer (Bettersize Instruments Ltd., Dandong, China), and the dispersion was then stored at 4 °C until use.

### 4.3. Pork–Whole Soy Milk Complex Gel (PSG) Preparation

Minced pork and whole soy milk were blended at a ratio of 3:7 (*w*/*w*). XG, KGM, and GG were incorporated at concentrations of 0.5%, 1.0%, and 1.5% (*w*/*w*), respectively. The mixture was homogenized at 10,000 r/min for 40 s using the HM6300 homogenizer (LabPrecision Beijing Technology Co., Ltd., Beijing, China). Aliquots of 100 g were transferred to 200 mL beakers, sealed with polyethylene film, incubated at 40 °C for 30 min, and then heated at 90 °C for 30 min to complete gelation. The gels were equilibrated at 4 °C for 12 h prior to analysis.

### 4.4. Determination of Cooking Loss and Water-Holding Capacity

Determine the cooking loss according to the method of Dong et al. [39]. Weigh the sample before heating. After the two-stage heating process, gently absorb the surface moisture from the composite gel using filter paper, then weigh it again. The cooking loss is calculated as follows:

(1) $$Cooking\;loss=\frac{m_{1}-m_{2}}{m_{1}}\times 100\%$$

where *m*_1_ and *m*_2_ represent the weights (g) of the composite gel before and after heating, respectively.

The water-holding capacity (WHC) was determined using the method described by Yu et al. [40] with slight modifications. Balance the composite gel at 25 °C and cut it into approximately 5 mm thick pieces. Weigh each piece, wrap it in double-layer filter paper, and put it into a centrifuge tube. Centrifuge the tubes at 4 °C and 10,000× *g* for 15 min. Following centrifugation, remove the composite gel sample, carefully blot the exuded water, and reweigh it. The WHC is calculated as follows:

(2) $$WHC=\frac{m_{4}}{m_{3}}\times 100\%$$

where *m*_3_ and *m*_4_ represent the weights (g) of the composite gel before and after centrifugation, respectively.

### 4.5. LF-NMR Relaxation Behavior

Wrap 5 g of PSG sample with polyethylene film and inserted into the 25 mm probe of a low-field NMR spectrometer (NMI20-040H-I, Niumag Co., Ltd., Suzhou, China). Use the Carr–Purcell–Meiboom–Gill sequence to measure the transverse relaxation time (T2), with the following measurement parameters: a radiofrequency (RF) delay of 0.002 ms, 140 echoes, a repetition time (TR) of 1000 ms, and 4 scans.

### 4.6. Color Measurement

The color difference in the composite gels was analyzed using a colorimeter. Prior to measurement, the colorimeter (CR-400, Konica Minolta Inc., Tokyo, Japan) was calibrated according to the protocol described by Pematilleke et al. [28]. Color measurements were obtained from three randomly selected locations on the surface of each gel. The measured parameters included L* (lightness), a* (red/green), and b* (yellow/blue). The whiteness value was calculated using the following Formula (3). The total color difference (∆E) between each sample and the control was determined according to the following Formula (4):

(3) $$Whiteness=100-\sqrt{{\left(100-L^{*}\right)}^{2}+{\left(a^{*}\right)}^{2}+{\left(b^{*}\right)}^{2}}$$

(4) $$\Delta E=\sqrt{{\left(\Delta L^{*}\right)}^{2}+{\left(\Delta a^{*}\right)}^{2}+{\left(\Delta b^{*}\right)}^{2}}$$

### 4.7. Texture Measurement and IDDSI Test

Prior to testing, the PSGs were equilibrated at 25 °C for 2 h. Texture profile analysis of the gels was then performed using a TA-XT Plus texture analyzer (Stable Micro Systems, Godalming, UK). Samples were loaded into a standard test cup (40 mm in diameter, 20 mm in depth) and filled to the marked line at a height of 15 mm. Compression tests were conducted using a dedicated cylindrical probe P/20 with a diameter of 20 mm. The initial height of the probe was set to 20 mm. The test speed was 10.0 mm/s, the post-test return speed was 10.0 mm/s, and the compression distance was 15 mm. The experiment was performed with five replicates.

The PSGs were cut into small pieces of 1.5 cm × 1.5 cm × 0.75 cm size according to the IDDSI test method. The fork pressure test is to press the gel sample with a fork with the thumb until the thumb nail becomes white (pressure is about 17 Kpa). This pressure is consistent with the force of the tongue when swallowing. Deformation was compared against IDDSI descriptors to assign texture level [41].

### 4.8. Rheological Characterization

The rheological properties of PSG were evaluated using an AR1500EX rheometer (DHR-1, TA Instruments, New Castle, DE, USA) equipped with a 40 mm parallel plate fixture (test gap 1 mm).

Frequency Sweep: Frequency sweep tests were conducted at a constant temperature of 25 °C to measure the storage modulus (G′) and loss modulus (G″) over an angular frequency range of 0.1–10 Hz, with a fixed strain amplitude of 0.5%.

Temperature sweep: Prior to each test, the sample was uniformly applied to the bottom plate. A thin layer of silicone oil was applied to the exposed edges to minimize moisture loss during measurement. After a 5 min equilibration at 25 °C, the following temperature sequence was applied: heating from 25 °C to 90 °C at 5 °C/min, holding at 90 °C for 30 min, cooling to 25 °C at 5 °C/min, and a final isothermal hold at 25 °C for 15 min. The oscillatory mode was maintained throughout the temperature program with a fixed strain of 0.5% and a frequency of 1 Hz.

### 4.9. Protein Interaction Analysis

According to the protocol established by the laboratory [35], the molecular forces within PSG were characterized using a chemical extraction method. The samples were dispersed sequentially in four different buffer solutions, each designed to dissolve specific molecular interactions: electrostatic interactions (*ES*), hydrophobic interactions (*H_y_*), disulfide bonds (*SS*), and hydrogen bonds (Hb). The buffer compositions were as follows: *B*_1_ (*ES*): 50 mM sodium phosphate buffer. *B*_2_ (*ES* + *H_y_*): 50 mM sodium phosphate buffer + 0.2% SDS. *B*_3_ (*ES* + *H_y_* + *SS*): 50 mM sodium phosphate buffer + 0.2% SDS + 20 mM TCEP hydrochloride. *B*_4_ (*ES* + *Hb*): 50 mM sodium phosphate buffer + 1.5 M urea. All solutions were maintained at pH 7.5.

(5) $$C_{n,bond,B_{i}}=\frac{\left(m_{s}+m_{gel}\right)}{m_{ge}l}\times C_{n,sup,B_{i}}$$

(6) $$P\left(ES\right)=\frac{C_{n,bond,B_{1}}}{C_{n,gel}}$$

(7) $$P\left(Hb\right)=\frac{C_{n,Bond,B_{2}}}{C_{n,gel}}-\frac{C_{n,Bond,B_{1}}}{C_{n,gel}}$$

(8) $$P\left(Hy\right)=\frac{C_{n,bond,B_{3}}}{C_{n,gel}}-\frac{C_{n,bond,B_{1}}}{C_{n,gel}}$$

(9) $$P\left(SS\right)=\frac{C_{n,bond,B_{4}}}{C_{n,gel}}-\frac{C_{n,gel,B_{3}}}{C_{n,gel}}$$

Among these variables, *m_s_* represents the mass of the buffer, *m_gel_* represents the mass of PSGs, *C_n_*, *gel* represent the total protein content of PSGs, and *C_n_*, bond, *B*_x_ represent the protein content in the supernatant obtained with the corresponding buffer.

### 4.10. Fourier Transform Infrared (FTIR)

Following Min et al. [42], the gels were frozen at −80 °C for 24 h and lyophilized. The dried material was ground under desiccation, mixed with KBr (1:100, *w*/*w*), and pressed into a 13 mm translucent disk. Spectra were collected using an IRTracer-100 spectrometer (Shimadzu Corporation, Kyoto, Japan) over the range of 400–4000 cm^−1^ (4 cm^−1^ resolution, 32 scans).

### 4.11. Scanning Electron Microscopy (SEM)

The microstructure of the composite gel was observed using SEM (S-3400N, Hitachi High-Technologies Corporation, Tokyo, Japan) [43]. The sample was initially frozen in liquid nitrogen and subsequently freeze-dried. The freeze-dried PSG sample was then sectioned along the cross-section, mounted on an aluminum stub, and sputter-coated with gold prior to imaging. SEM images were acquired at an acceleration voltage of 5 kV.

### 4.12. Statistical Analysis

All samples were analyzed in triplicate, with results presented as mean ± standard deviation (SD). Statistical analysis was performed using SPSS software (version 27; IBM Corporation, Armonk, NY, USA) for one-way analysis of variance (ANOVA) followed by Duncan’s multiple range test. A significance level of *p* < 0.05 was applied.

## Figures and Tables

**Table 1:** Color coordinates of the PSGs composite gels at different polysaccharide addition levels.

Sample	L*	a*	b*	Whiteness	ΔE
CK	80.59 ± 0.32 ^b^	4.14 ± 0.07 ^a^	9.58 ± 0.76 ^bc^	77.96 ± 0.36 ^b^	0
XG 0.5%	80.70 ± 0.24 ^b^	3.72 ± 0.07 ^ef^	9.10 ± 0.41 ^cd^	78.33 ± 0.22 ^b^	0.64
XG 1.0%	81.75 ± 0.25 ^ab^	3.80 ± 0.20 ^def^	8.72 ± 0.18 ^de^	79.42 ± 0.30 ^ab^	1.46
XG 1.5%	83.09 ± 0.21 ^a^	3.72 ± 0.03 ^ef^	8.36 ± 0.12 ^e^	80.78 ± 0.18 ^a^	2.79
KGM 0.5%	81.80 ± 0.95 ^ab^	4.07 ± 0.05 ^ab^	10.00 ± 0.24 ^ab^	78.84 ± 0.91 ^ab^	1.29
KGM 1.0%	81.49 ± 0.95 ^ab^	4.00 ± 0.14 ^abc^	9.47 ± 0.24 ^bc^	78.82 ± 0.81 ^ab^	0.92
KGM 1.5%	80.53 ± 0.36 ^b^	3.92 ± 0.10 ^bcd^	9.56 ± 0.21 ^bc^	77. 95 ± 0.35 ^b^	0.23
GG 0.5%	82.09 ± 0.70 ^ab^	3.88 ± 0.07 ^cde^	10.20 ± 0.42 ^a^	79.02 ± 0.51 ^ab^	1.64
GG 1.0%	81.37 ± 0.33 ^ab^	3.80 ± 0.15 ^def^	9.96 ± 0.06 ^ab^	78.84 ± 0.28 ^ab^	0.94
GG 1.5%	81.05 ± 0.58 ^ab^	3.62 ± 0.13 ^f^	9.79 ± 0.32 ^ab^	78.80 ± 0.90 ^ab^	0.72

Means that the different superscript letters within the same column for each parameter are significantly different (*p* < 0.05).

**Table 2:** Textural properties of different formulations of PSGs.

Sample	Hardness (×10^4^ N/m^2^)	Adhesion (×−10^3^ J/m^3^)	Agglomeration
CK	3.15 ± 0.10 ^a^	2.51 ± 0.50 ^e^	0.417 ± 0.005 ^e^
XG 0.5%	1.34 ± 0.23 ^d^	1.80 ± 0.48 ^c^	0.459 ± 0.029 ^cde^
XG 1.0%	0.74 ± 0.02 ^f^	1.22 ± 0.21 ^ab^	0.499 ± 0.036 ^bc^
XG 1.5%	0.70 ± 0.02 ^f^	1.01 ± 0.23 ^a^	0.508 ± 0.016 ^b^
KGM 0.5%	1.59 ± 0.14 ^c^	2.87 ± 0.30 ^e^	0.461 ± 0.021 ^cd^
KGM 1.0%	1.34 ± 0.10 ^d^	2.44 ± 0.45 ^e^	0.539 ± 0.022 ^b^
KGM 1.5%	1.29 ± 0.17 ^de^	2.37 ± 0.38 ^de^	0.591 ± 0.049 ^a^
GG 0.5%	1.81 ± 0.25 ^b^	1.54 ± 0.65 ^bc^	0.442 ± 0.039 ^de^
GG 1.0%	1.26 ± 0.05 ^de^	1.87 ± 0.22 ^cd^	0.461 ± 0.007 ^cd^
GG 1.5%	1.10 ± 0.07 ^e^	1.90 ± 0.17 ^cd^	0.502 ± 0.08 ^bc^

Means that the different superscript letters within the same column for each parameter are significantly different (*p* < 0.05).

**Table 3:** IDDSI results of different formulations of PSGs.

Sample	Fork Pressure Test	Comments	Sample	Fork Pressure Test	Comments
CK	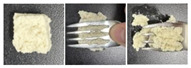	×	KGM 1.0%	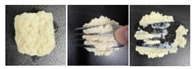	Level 6
XG 0.5%	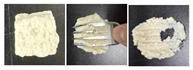	Level 6	KGM 1.5%	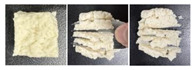	Level 6
XG 1.0%	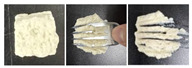	Level 5	GG 0.5%	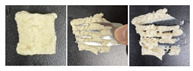	Level 6
XG 1.5%	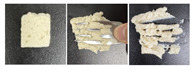	Level 5	GG 1.0%	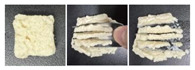	Level 6
KGM 0.5%	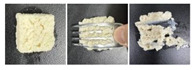	Level 6	GG 1.5%	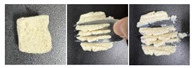	Level 6

**Table 4:** Modeling of PSGs rheological behavior; gel temperature from temperature sweep and G25 °C/G90 °C ratio.

	Model: log(G’) = n × log(ω) + K				
Sample	n	K	R^2^	Gel Temperature(°C)	G′_25°C_/G′_90°C_
CK	0.0569 ± 0.0026 ^f^	4.7201 ± 0.0137 ^a^	0.9786 ± 0.0034	45.73 ± 2.51 ^a^	2.75 ± 0.15 ^a^
XG 0.5%	0.0643 ± 0.0006 ^de^	3.7878 ± 0.0237 ^cd^	0.9810 ± 0.0075	45.93 ± 2.70 ^ab^	2.45 ± 0.19 ^ab^
XG 1.0%	0.0631 ± 0.0005 ^e^	3.4206 ± 0.0349 ^g^	0.9860 ± 0.0012	47.44 ± 2.44 ^ab^	2.06 ± 0.22 ^c^
XG 1.5%	0.0621 ± 0.0009 ^e^	3.1844 ± 0.0662 ^h^	0.9919 ± 0.0023	47.49 ± 1.08 ^ab^	2.11 ± 0.17 ^c^
KGM 0.5%	0.0668 ± 0.0021 ^d^	3.7469 ± 0.0079 ^de^	0.9934 ± 0.0009	47.29 ± 2.05 ^ab^	2.46 ± 0.23 ^ab^
KGM 1.0%	0.0769 ± 0.0008 ^b^	3.7771 ± 0.0154 ^cd^	0.9855 ± 0.0008	49.16 ± 2.79 ^b^	2.29 ± 0.13 ^bc^
KGM 1.5%	0.0881 ± 0.0020 ^a^	3.7192 ± 0.0072 ^e^	0.9842 ± 0.0021	53.41 ± 1.98 ^a^	2.76 ± 0.28 ^a^
GG 0.5%	0.0649 ± 0.0012 ^de^	4.0624 ± 0.0249 ^b^	0.9718 ± 0.0101	47.74 ± 1.77 ^ab^	2.35 ± 0.08 ^bc^
GG 1.0%	0.0698 ± 0.0016 ^c^	3.8072 ± 0.0027 ^c^	0.9824 ± 0.0045	48.75 ± 1.79 ^ab^	2.54 ± 0.24 ^ab^
GG 1.5%	0.0725 ± 0.0029 ^c^	3.6192 ± 0.0090 ^f^	0.9828 ± 0.0090	48.21 ± 3.71 ^ab^	2.51 ± 0.21 ^ab^

Means that the different superscript letters within the same column for each parameter are significantly different (*p* < 0.05).

## Data Availability

The original contributions presented in this study are included in the article. Further inquiries can be directed to the corresponding authors.
